# Translesion Synthesis: Insights into the Selection and Switching of DNA Polymerases

**DOI:** 10.3390/genes8010024

**Published:** 2017-01-10

**Authors:** Linlin Zhao, M. Todd Washington

**Affiliations:** 1 Department of Chemistry and Biochemistry, Central Michigan University, Mount Pleasant, MI 48859, USA; 2 Science of Advanced Materials Program, Central Michigan University, Mount Pleasant, MI 48859, USA; 3 Department of Biochemistry, Carver College of Medicine, University of Iowa, Iowa City, IA 52242, USA; todd-washington@uiowa.edu

**Keywords:** DNA damage, DNA lesion bypass, DNA polymerase, genomic instability, mutagenesis, translesion synthesis

## Abstract

DNA replication is constantly challenged by DNA lesions, noncanonical DNA structures and difficult-to-replicate DNA sequences. Two major strategies to rescue a stalled replication fork and to ensure continuous DNA synthesis are: (1) template switching and recombination-dependent DNA synthesis; and (2) translesion synthesis (TLS) using specialized DNA polymerases to perform nucleotide incorporation opposite DNA lesions. The former pathway is mainly error-free, and the latter is error-prone and a major source of mutagenesis. An accepted model of translesion synthesis involves DNA polymerase switching steps between a replicative DNA polymerase and one or more TLS DNA polymerases. The mechanisms that govern the selection and exchange of specialized DNA polymerases for a given DNA lesion are not well understood. In this review, recent studies concerning the mechanisms of selection and switching of DNA polymerases in eukaryotic systems are summarized.

## 1. Introduction

DNA is susceptible to numerous endogenous and exogenous chemicals, producing a wide variety of DNA lesions. Unrepaired DNA lesions are potential sources of replication and transcription errors, replication fork arrest, and cell death, which together contribute to genomic instability and pathogenesis. Two strategies exist to counteract replication fork stalling. One involves template switching, in which the undamaged template from the sister chromatid is used for recombination-dependent DNA synthesis; this process is usually error-free. A second strategy is to use one or more of the translesion synthesis (TLS) DNA polymerases (pols) to accomplish nucleotide incorporation opposite and past the DNA lesion before a replicative DNA polymerase (pol ε or pol δ in eukaryotes) resumes its function. This process—which is intrinsically error-prone—is a major source of DNA damage-induced mutagenesis [1].

Genetic studies in the 1970s showed that mutations in the UV nonmutable (*umu*) locus in *Escherichia coli* (*E. coli*) [2,3] and the reversionless (*REV*) locus in *Saccharomyces cerevisiae* (*S. cerevisiae*) [4,5] were associated with deficiencies in mutagenesis in these organisms upon treatment with DNA-damaging agents. Around the same time, cells from patients with a variant form of a cancer predisposition syndrome *xeroderma pigmentosum* (XP-V) were found to be deficient in synthesizing daughter DNA strands after UV irradiation [6]. It was not until the 1990s that the products of these and related genes were purified and biochemically characterized. The product of the yeast *REV1* gene was found to be a dCMP transferase [7], and the product of the yeast *REV3* gene was shown to be the catalytic subunit of pol ζ, which is able to bypass a common UV-induced cyclobutane pyrimidine dimer (CPD) DNA lesion with low efficiency [8]. In 1999, the yeast Rad30 protein was shown to be able to replicate past a thymine–thymine CPD as efficiently and accurately as with undamaged thymines [9]. Shortly after, defects in the human gene encoding Rad30 was shown to cause the XP-V syndrome [10,11]. By 2000, the arsenal of TLS polymerases had expanded rapidly with the discovery of *E. coli* pol IV (DinB) [12] and pol V (UmuC) [13,14], pol ι (a second human ortholog of Rad30) [15,16,17,18], and pol κ (a human ortholog of *E. coli* DinB) [19,20,21,22]. These findings led to the realization that TLS is a conserved process from bacteria to humans [23], which involves a large family of proteins, known as TLS DNA polymerases.

Today, 17 human DNA polymerases have been purified and biochemically characterized, and these proteins are classified into A, B, X, Y, and AEP (archaeo-eukaryotic primase superfamily) families according to their sequence homology and structural similarities [24,25,26]. The best-characterized Y-family DNA polymerases include pol η, pol ι, pol κ, and Rev1, which, together with B-family enzyme pol ζ, are the principle TLS pols in humans. Pols of A and X families also have TLS activities and contribute to mutagenesis in DNA repair pathways such as base excision repair and non-homologous end joining (NHEJ) [27]. The most recently discovered DNA polymerase/primase PrimPol (AEP superfamily) has the capability of bypassing a number of DNA lesions [26,28,29,30,31]. More importantly, PrimPol has primase activity that can perform de novo DNA synthesis using deoxyribonucleotide triphosphates (dNTPs), which is important for replication re-start downstream of a stalled fork [32,33,34,35]. Nowadays, the understanding of TLS polymerases has evolved from their conventional lesion bypass activities to myriad roles in organismal fitness and disease, such as to increase the diversity of the immunoglobulin gene during hypermutation, to overcome secondary DNA structures during DNA copying, to participate in DNA repair, and to contribute to mutagenesis in tumors [25,27,36,37].

Translesion synthesis is thought to occur via two non-mutually exclusive processes. One is for TLS pols to participate at a replication fork, and the other is to fill post-replicative gaps [38]. The first process involves several polymerase-switching processes, including dissociation of a stalled replicative polymerase from the replication fork, binding of one or two TLS polymerases to the replication terminus for nucleotide insertion and extension, and eventually displacement of TLS pols with a replicative polymerase downstream of the DNA lesion [38,39]. The latter pathway requires fewer switching events. A major unanswered question is how polymerase switching occurs at the replication factories (reviewed in [40,41,42]). Deciphering the mechanisms of the polymerase exchange is not only fundamental for the understanding of translesion synthesis, but also important for the development of chemotherapy to control TLS activities [25,38,43]. This is because many cancer chemotherapies work by damaging DNA, and inhibiting TLS pols that affect DNA repair capability holds promise for improving responses to treatments [25,43]. This review aims to summarize recent studies on the mechanistic aspects of TLS in eukaryotic systems. For detailed discussions on the biochemical properties, regulation, and functions of TLS DNA polymerases, please see these excellent reviews [24,27,38,44,45,46]. Readers interested in TLS in bacteria are referred to the following reviews [42,47].

## 2. Selection and Switching of Specialized DNA Polymerases

DNA is susceptible to a variety of chemicals from endogenous and exogenous sources, which generates up to 100,000 DNA lesions per cell each day [48]. Selection of the most appropriate specialized DNA polymerase to bypass a given lesion is dictated by a number of possible factors. One obvious factor is the identity of DNA lesions. A second potential factor is the interactions of specialized polymerases with hub proteins such as PCNA and Rev1. Other potential factors include the availability of TLS polymerases in the vicinity of stalled replication forks owing to cell cycle and transcription regulation or protein degradation.

### 2.1. Selection of the “Right” TLS Pol for Benzo[a]pyrene-Derived DNA Lesions: A Case Study

In eukaryotes, various TLS polymerases have evolved to accommodate different types of DNA damage. When a polymerase is recruited to a stalled fork, it can only be used if it is able to accommodate the damaged primer-template in its active site and is able to catalyze the nucleotide-incorporation reaction [38]. Certain DNA modifications can be bypassed by replicative DNA polymerases [49,50], whereas bulky DNA lesions, such as carcinogen benzo[*a*]pyrene (BaP)-derived DNA damage, often require one or more TLS DNA polymerases to facilitate the fork progression [51]. Knowledge concerning cognate DNA lesions of each TLS pol has been reviewed [27,44,52]. TLS pols often act redundantly in the bypass of a given DNA lesion, and it is challenging to firmly identify the most biologically relevant DNA lesion for some pols. A few structurally distinct DNA lesions, such as cyclobutane pyrimidine dimer (CPD) and BaP-derived lesions, require specific polymerase activities [53,54]. Multiple factors including the chemistry of DNA lesion and DNA polymerase structure affect the selection of TLS pols. In the following section, DNA lesions derived from BaP, a prototypical carcinogen, will be used as an example to discuss how the chemistry of DNA lesions affects the enzymatic activities of DNA polymerases.

#### 2.1.1. BaP-Induced DNA Damage

**Figure 1:**
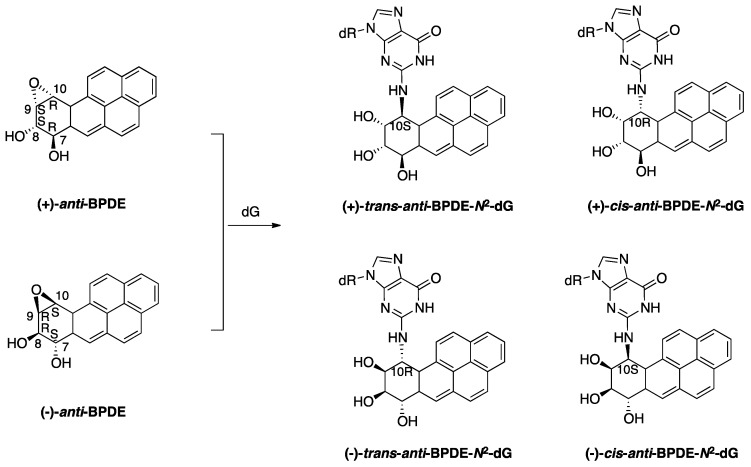
Structures of stereoisomers of BPDE-derived *N*^2^-dG DNA adducts.

BaP is a ubiquitous environmental pollutant that exists in overcooked meat, vehicular exhaust, coal tar, and tobacco smoke. BaP is a Group 1 carcinogen classified by the International Agency for Research on Cancer (IARC), and has been associated with skin, lung, and colon cancers in humans [55,56]. The carcinogenicity of BaP is attributed in part to its ability to form the ultimate tumorigenic metabolites (+)-7*β*,8*α*-dihydroxy-9*α*,10*α*-epoxy-7,8,9,10-tetrahydrobenzo[*a*]pyrene [7R,8S,9S,10R steric configuration; the most distant hydroxyl group is *anti* relative to the orientation of the epoxide group, and is hereinafter referred to as (+)-*anti*-BPDE] and (−)-7*α*,8*β*-dihydroxy-9*β*,10*β*-epoxy-7,8,9,10-tetrahydrobenzo[*a*]pyrene [7S,8R,9R,10S steric configuration; (−)-*anti*-BPDE] (Figure 1). (+)-*anti*-BPDE is more tumorigenic than its enantiomer (−)-*anti*-BPDE [57,58,59]. Both metabolites react with the *N*^2^ exocyclic amino group of guanine (Figure 1) and to a lesser extent with the *N*^6^ exocyclic amino groups of adenine and the *N*^4^ exocyclic amino groups of cytosine to form DNA adducts [60,61]. Due to the carcinogenic potency, BPDE-derived DNA lesions are among the best-studied DNA lesions in terms of their toxicological mechanisms. Alternative bioactivation routes can convert BaP to radical cations that are reactive towards the C8 or N7 atoms of guanine and the N3 or N7 positions of adenine, some of which can form mutagenic apuridinic/apyrimidinic (AP) sites due to the unstable glycosidic linkage [62]. Other pathways involve biotransformation via aldo-keto reductase to yield reactive quinone-derived DNA adducts that are chemically labile or stable [63,64]. BaP-derived DNA lesions block DNA synthesis by replicative pols and induce mutagenic replication products via TLS. A prevalent mutation resulting from BaP exposure is a G to T transversion, a common mutation found in BaP-treated mammalian cells and the *p53* gene of lung cancers of smokers [55,56]. The local sequence context of BaP-induced DNA damage also plays a role in the resulting mutation pattern [65,66,67,68].

#### 2.1.2. Accurate Bypass of BaP-Derived DNA Lesions

Major BPDE-derived DNA lesions include the stereoisomeric 2′-deoxyguanine (dG) adducts (+)-*trans*-*anti*-BPDE-*N*^2^-dG, (+)-*cis*-*anti*-BPDE-*N*^2^-dG, (−)-*trans*-*anti*-BPDE-*N*^2^-dG and (−)-*cis*-*anti*-BPDE-*N*^2^-dG (Figure 1), as well as the 2′-deoxyadenosine (dA) adducts (+)-*trans*-*anti*-BPDE-*N*^6^-dA, (+)-*cis*-*anti*-BPDE-*N*^6^-dA, (−)-*trans*-*anti*-BPDE-*N*^6^-dA and (−)-*cis*-*anti*-BPDE-*N*^6^-dA. These lesions are able to assume a variety of conformations depending on the local sequence context, as evidenced by solution nuclear magnetic resonance (NMR) structures (reviewed in [69] and references therein). Consequently, there is no universal TLS pol to bypass all lesions due to their structural diversity and the varying bypass capabilities of TLS pols. In addition, effects of the host cell and the local sequence context contribute to the varying degrees of bypass efficiencies and the resulting mutations [70]. Pol κ is well known for its role in the accurate bypass of BPDE-*N*^2^-dG DNA lesions. Pol κ is capable of replicating past all four BPDE-derived *N*^2^-dG lesions in a primarily error-free fashion in vitro and in vivo [22,71,72,73,74] and is protective against the mutagenic effects of BaP in cells [54,75]. However, pol κ is unable to bypass (+)-*trans*-*anti*-BPDE-*N*^6^-dA or (−)-*trans*-*anti*-BPDE-*N*^6^-dA lesions [76], and these lesions are thought to contribute to the mutagenicity of low-dose BaP exposure [77,78,79]. The extent of the involvement of pol κ in the accurate replication across the (+)-*trans*-*anti*-BPDE-*N*^2^-dG lesion in cells remains controversial, mostly likely due to the different sequences and cell lines used in respective laboratories. Using a quantitative bypass assay, Avkin et al. demonstrated that approximately 60% of the (+)-*trans*-*anti*-BPDE-*N*^2^-dG adducts require pol κ for accurate bypass [75]. On the other hand, Hashimoto et al. showed that the error-free products account for less than 10% of total TLS products with the same DNA lesion in mouse embryonic fibroblasts [80]. Pol ι, which is the least accurate TLS pol, is known for preferentially misincorporating T opposite unmodified G [81]. Interestingly, in vitro pol ι incorporates a correct nucleotide opposite stereoisomeric BPDE-*N*^2^-dA adducts, although it is unable to insert nucleotides opposite BPDE-*N*^2^-dG adducts or to extend the primer beyond the lesion [76,82]. Further experiments are needed to confirm the biological significance of this particular bypass activity of pol ι.

**Figure 2:**
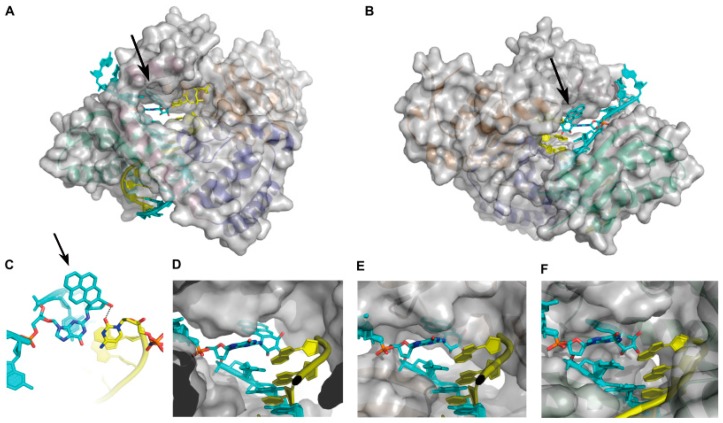
The structures of DNA polymerase complexes with a BPDE-dG lesion-containing duplex. The adducted template is shown in cyan, and the primer and incoming dCTP are shown in yellow. The black arrows are pointing at the BPDE ring. (**A**,**B**) Different views of the X-ray crystal structure of pol κ:(+)-*trans*-dG-*N*^2^-BPDE-DNA:dCTP (pol κ-BPDE) complex (PDB: 4U7C). The major groove of DNA is facing the viewer in (**A**); and the minor groove of DNA is facing the viewer in (**B**). (**C**) Base pairing of (+)-*trans*-dG-*N*^2^-BPDE lesion and the incoming dCTP at the active site of pol κ. An additional hydrogen bond formed between a hydroxyl group of BPDE and the O_2_ atom of cytidine is shown with a dashed line. (**D**) Zoomed-in view of pol κ accommodating the BPDE ring in an open DNA binding cleft. (**E**) Structural model of pol ι (PDB: 4FS2) with an adducted substrate. The conformation of the DNA is adopted from the pol κ-BPDE structure. (**F**) Structural model of pol η (PDB: 3MR2) with an adducted substrate. The conformation of the DNA is from the pol κ-BPDE structure. For simplicity, the incoming dCTP is omitted in (**D**–**F**).

A recent X-ray crystal structure of pol κ:(+)-*trans*-*anti*-BPDE-*N*^2^-dG-DNA:dCTP (pol κ-BPDE) complex has provided insights into why pol κ is adept at bypassing bulky BPDE-induced DNA lesions [83]. The overall structure of pol κ-BPDE closely resembles the structure of a pol κ complex with an unmodified DNA substrate, indicating that pol κ accommodates the (+)-*trans*-*anti*-BPDE-*N*^2^-dG lesion at the active site (Figure 2A,B). The BPDE-adduced substrate adopts a standard B-form of DNA, and the BPDE-*N*^2^-dG adduct retains the *anti* conformation. The BPDE ring is positioned in the minor groove and forms an additional H-bond with the incoming dCTP (Figure 2C). The BPDE ring points towards the 5′-end of the template strand, consistent with the solution NMR structures of DNA containing BPDE-derived dG lesions [84,85]. This conformation of the adduct is accommodated by an open DNA binding cleft in pol κ (Figure 2D), which is not found in pol η or pol ι. Modeling this conformation of the BPDE-adduced DNA into the structures of pol η (Figure 2E) and pol ι (Figure 2F) results in steric clash with both pols. In addition, the unique N-clasp domain of pol κ (not found in other Y-family TLS pols) supports an open conformation of the protein and stabilizes the single-stranded template for the efficient and error-free bypass of BPDE-dG DNA lesions [83].

The fact that pol ι is able to incorporate the correct dTTP opposite BPDE-*N*^6^-dA DNA lesions in vitro suggests that pol ι can accommodate certain conformers of BPDE-*N*^6^-dA DNA lesions at the active site. Although a ternary structure of pol ι with the BPDE-*N*^6^-dA lesion and an incoming nucleotide is unavailable, molecular dynamics simulations have demonstrated that a BPDE-*N*^6^-dA lesion assumes an *anti* or *syn* conformation at the active site of pol ι depending on the adjacent nucleotides forming a Watson–Crick or Hoogsteen base pair with the incoming dTTP, respectively [86]. The BPDE ring is positioned in the major groove due to the relatively narrow active site of pol ι, and forms additional H-bonds with nearby nucleotides [86].

#### 2.1.3. Error-Prone Bypass of BaP-Derived DNA Lesions

BaP-induced mutations are fueled at least in part by error-prone DNA replication across BPDE-derived DNA adducts [87]. More than 90% of the bypass events across the (+)-*trans*-*anti*-BPDE-*N*^2^-dG DNA lesion are error-prone in mouse embryonic fibroblasts [80]. For pol η, DNA synthesis is almost completely blocked by (−)-*trans*-*anti*-BPDE-*N*^6^-dA adduct, whereas weak and error-prone bypass activities exist for both stereoisomeric BPDE-*N*^2^-dG adducts and (+)-*trans*-*anti*-BPDE-*N*^6^-dA adduct [76]. Using human XP-V fibroblasts that express a truncated and non-functional pol η [9], Avkin et al. found that the bypass of the (+)-*trans*-*anti*-BPDE-*N*^2^-dG-DNA lesion is largely accurate and concluded that pol η is not essential for TLS across this particular lesion with the template sequence they used [75]. On the other hand, pol ζ plays an important role in the mutagenic bypass of the (+)-*trans*-*anti*-BPDE-*N*^2^-dG-DNA lesion, which is likely due to its function as an extender DNA polymerase [51,80]. The importance of pol ζ in error-free bypass of BPDE-derived lesions remains controversial [51,80]. Rev1 is known for its deoxycytidyl transferase activity and its role as a scaffold protein to interact with other Y-family DNA polymerases [7,88,89,90,91,92,93]. Although Rev1 is capable of inserting dCTP opposite (+)-*trans*-*anti*-BPDE-*N*^2^-dG and (−)-*trans*-*anti*-BPDE-*N*^2^-dG DNA lesions in vitro [88], its role in error-free bypass seems to be nonessential in mouse cells [80]. Instead, the non-catalytic function of Rev1 is important for pol κ-mediated BPDE resistance of mouse embryonic fibroblast cells [94], and for the erroneous bypass of the (+)-*trans*-*anti*-BPDE-*N*^2^-dG lesion by pol ζ [80].

Together, it is apparent that the identity of BaP-derived DNA lesions drives the selection of TLS pols. Multiple factors, including the steric effects, tautomerization, the ability to form base pairs with the incoming nucleotide and local sequence context, seem to affect the selection of TLS pols. Apart from BaP-derived DNA lesions, a variety of DNA lesions have been assayed in vitro and in cellular experiments to identify the most biologically relevant TLS pol(s); however, in many cases, different TLS pols act redundantly during TLS [52], and it remains a challenge to generate a list of cognate lesions for each TLS pol. It seems logical for backup enzymes to exist for DNA replication and repair. The fact that a respective TLS pol has evolved to protect against the mutagenic effects of BPDE and CPD-derived DNA damage underscores the importance of these carcinogens.

### 2.2. PCNA: An Interaction Hub for Many Partners

PCNA is known for orchestrating a variety of components in DNA metabolism. PCNA was first discovered as an auxiliary protein that stimulates the activity of DNA polymerase δ [95,96], and was subsequently recognized for its remarkable abilities in coordinating multiple cellar processes such as unperturbed DNA replication, translesion synthesis, Okazaki fragment maturation, DNA repair, chromatin remodeling, and cell cycle regulation [97,98,99,100]. PCNA promotes the access of specialized pols to the replication factories through physical and functional interactions with these proteins. PCNA interacts with purified Y-family TLS pols and stimulates the catalytic efficiencies of these polymerases in vitro [101,102,103,104]. The understanding of the importance of these interactions in vivo was obtained primarily from nuclear focus-formation assays with DNA damaging reagent-treated cells ([105] and references therein). However, care should be taken in interpreting these results because the composition of these foci and whether they represent direct interactions are not known [105]. In this section, the biochemical basis of interactions between PCNA and different DNA polymerases is discussed.

#### 2.2.1. Interactions between PCNA and DNA Polymerases

**Figure 3:**
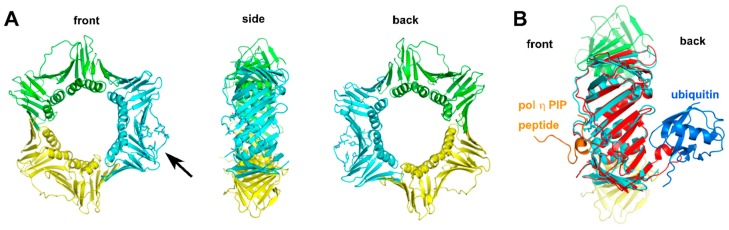
Structures of human PCNA and yeast ubiquitinated PCNA. (**A**) Front, side, and back views of human PCNA (PDB:2ZVK). Three subunits are shown in green, yellow, and cyan. In one subunit (cyan), amino acid residues surrounding the hydrophobic pocket near the interdomain-connecting loop are shown in stick. The black arrow is pointing at the hydrophobic pocket. For simplicity, the pol η PIP peptide is omitted from the original crystal structure. (**B**) A subunit of yeast ubiquitinated PCNA (red; PDB:3L10) is superimposed with a subunit (cyan) of human PCNA (PDB:2ZVK). The pol η PIP peptide (orange) interacts with the hydrophobic pocket on the front side of PCNA, and ubiquitin (blue) interacts with the back side of PCNA.

Eukaryotic PCNA comprises three identical subunits, and each subunit has two similarly folded domains joined by an interdomain connector loop (Figure 3A) [106,107]. The homotrimeric eukaryotic PCNA is assembled into a circular ring with a central hole that is wide enough to encircle the DNA and to allow diffusion of PCNA along the DNA [108]. The PCNA ring has one side facing the direction of DNA synthesis and the other side pointing away (hereinafter referred to as the front side and the back side of PCNA, respectively). The front side contains the C-terminus of each monomer and the interdomain-connecting loop. A hydrophobic pocket (Figure 3A) near the interdomain-connecting loop on the front side of each monomer serves as a platform to interact with DNA polymerases. In vitro, interactions between PCNA and purified TLS pols (e.g., human pol η, pol ι and pol κ) stimulate the catalytic efficiencies of these polymerases with unmodified and damaged DNA substrates via lowering the *K*_m_ of the incoming nucleotide [101,102,103,104]. Pol η and pol ι, but not pol κ, have elevated processivity in the presence of PCNA, replication factor C (RFC) and replication protein A (RPA) [101,102,103,104]. Pol ζ is stimulated by PCNA with lesion-bearing DNA, but not with unmodified substrates [109,110]. PCNA stimulates the catalytic efficiency of Rev1 and does so to a greater extent when the PCNA is monoubiquitinated [111].

#### 2.2.2. Biochemical Basis of PCNA-Pol Interactions

The interacting partners of PCNA in eukaryotes generally contain one or more PCNA-interacting protein (PIP) motifs. Based on the amino acid sequence of these motifs, PIPs are classified into canonical and non-canonical PIPs, which differ in their sequence and binding affinity for PCNA. Canonical PIPs, found in p21^WAF1/CIP1^ [107], the p66 subunit of pol δ [112] and FEN1 [113], have a consensus sequence Qxx[L/I/M]xx[F/Y][F/Y/W] featuring high-affinity interactions with PCNA. Non-canonical PIPs, on the other hand, have alternative residues at the first and last positions, lowering the binding affinity for PCNA relative to the consensus sequence. The difference in the binding affinities for PCNA potentially contributes to affinity-driven polymerase switching [98]. For example, the PIP peptide (QVSITGFF, canonical) of the p66 subunit of human pol δ has a higher affinity for PCNA relative to pol η (MQTLESFF, non-canonical) [114]. Changing the first amino acid residue of the PIP peptide of pol η to a glutamine (QQTLESFF) results in a four-fold increase in its affinity for PCNA [114]. The apparent dissociation constant (*K*_d_) of human pol δ, pol η, pol κ, and pol ι PIP peptides with PCNA are summarized in Table 1. Although affinity-driven competition has been proposed as a mechanism for polymerase switching, the molecular mechanism of this model remains to be studied in much detail. Rev1, on the other hand, has no PIP motifs, but interacts with PCNA through its N-terminal BRCA1 C-terminus (BRCT) domain [115,116] and/or polymerase-associated domain (PAD) [117]. This interaction between the PAD domain of Rev1 and PCNA observed in yeast remains to be confirmed in vertebrates. Importantly, several recent studies have discovered non-conventional interacting partners of the PIP motif as well as the related Rev1-interacting region (RIR, see below). For example, yeast pol η uses its PIP motif to interact with both PCNA and Rev1 [118], and human pol η uses one of its RIR motifs to interact with Rev1 and pol δ [119]. In fact, the very notion of a PIP motif as a distinct entity has recently been questioned, and it has been proposed that these and other related motifs be renamed PIP-like motifs to better reflect their broader roles in the network of interacting proteins responsible for DNA replication and repair [120].

#### 2.2.3. Ubiquitination of PCNA

Post-translational modifications of PCNA play an important role in DNA damage tolerance pathways [97,98,122]. Ubiquitination of PCNA, in particular, is known to participate in a variety of pathways during DNA replication and repair [122]. Ubiquitination of PCNA, mediated by the Rad6–Rad18 ubiquitination system, occurs in response to fork stalling near a lesion or an unusual DNA structure. Generally, the monoubiquitinated PCNA serves as an interacting platform for TLS DNA polymerases, whereas the polyubiquitinated PCNA is involved in error-free bypass via recombination-dependent pathways [122]. Ubiquitination of PCNA occurs primarily at K164 and to a lesser extent at other lysine residues [123,124]. One or two ubiquitin-binding motifs (UBMs; pol ι and Rev1) or ubiquitin-binding zinc-fingers (UBZs; pol η and pol κ) are present in Y-family DNA polymerases [125], which increase the affinity of DNA polymerases for monoubiquitinated PCNA and potentially facilitate the recruitment of TLS pols. In *S. cerevisiae*, it is established that the monoubiquitination of PCNA is essential for optimal TLS and TLS polymerase switching. For example, in vitro studies using recombinant yeast enzymes show that both unmodified and monoubiquitinated PCNA stimulates the efficiencies of nucleotide incorporation by pol η and REV1; however, a stronger stimulatory effect is observed when the PCNA is monoubiquitinated [111,126,127]. In addition, upon replication stalling, the exchange of yeast pol η and pol δ occurs in the presence of monoubiquitinated PCNA but not with the unmodified PCNA [128]. In yeast cells, Rad6-mediated monoubiquitination of PCNA is required to activate TLS by pol η [129,130].

On the contrary, in mammalian systems, whether a direct interaction between pol η and ubiquitinated PCNA is required (or even occurs) during TLS remains controversial. In human cells, UBMs are needed for foci formation of Y-family polymerases and for physical interactions between polymerases and ubiquitinated PCNA [125,131]. However, as mentioned earlier, the foci formation should not be used to conclude that a direct interaction between pol η and ubiquitinated PCNA is required (or even occurs) during TLS in mammalian systems. On the other hand, physical and specific interactions of pol η with ubiquitinated PCNA have been demonstrated with co-immunoprecipitation using cell extracts [132,133]. While Acharya et al. reported that a direct binding of the UBZ domain of pol η with ubiquitinated PCNA is not required during TLS [134], this conclusion has been questioned because the dispensability of the pol η UBZ domain is thought to be due to an artificially increased PCNA expression [135]. Other in vivo evidence suggests ubiquitination of PCNA is in fact dispensable. For example, pol η localizes into replication foci during unperturbed DNA replication [125] as well as upon treatment with UV irradiation [133,136,137] independently of PCNA monoubiquitination. Hendel et al. have shown that the ubiquitination of PCNA is important, but not essential for TLS in mouse cells [138]. Using photobleaching techniques, Sabbioneda et al. have demonstrated that PCNA ubiquitination is not required for the pol η foci formation, but increases the residence time of pol η in foci in human cells [136]. In addition, studies from several laboratories have demonstrated that PCNA ubiquitination is dispensable during lesion bypass [136,138,139], in which TLS pols may be recruited via interactions with Rev1 (discussed in Section 2.3) [140,141]. The interactions between TLS polymerases and PCNA are considered to be highly dynamic judging by the times of immobilization of pol η and pol ι (100–200 ms) upon DNA damage [136]. Therefore, it is proposed that pol η transiently and continually probes the exposed DNA for suitable substrates [136]. Recently, using quantitative kinetic assays and a reconstituted lagging-strand replication system, Hedglin et al. have shown that the binding of pol η to PCNA and pol η-catalyzed DNA synthesis occur without PCNA monoubiquitination, and that efficient exchange of pol η with pol δ happens owing to the intrinsic DNA binding properties of these pols [121]. Additional studies are warranted to unequivocally determine the biological functions of PCNA ubiquitination in vivo.

#### 2.2.4. Structure of Monoubiquitinated PCNA

The X-ray crystal structure of monoubiquitinated *S. cerevisiae* PCNA has provided additional insights into PCNA–polymerase interactions [126]. The expression of yeast monoubiquitinated PCNA is achieved by splitting the protein into two self-assembling polypeptides [126]. As shown in Figure 3B, the ubiquitin moiety uses its canonical hydrophobic surface to interact specifically but weakly with PCNA via electrostatic and hydrogen-bonding interactions. The attachment of ubiquitin does not alter the conformation of PCNA, suggesting that there is no or minimal conformational change of PCNA upon ubiquitin binding [126]. The ubiquitin molecule is located on the back side of PCNA, presumably leaving the hydrophobic pocket on the front side to interact with the PIPs of other DNA polymerases, which is consistent with a tool belt model of translesion synthesis. A PCNA tool belt is a structure with multiple TLS polymerases directly interacting with PCNA without directly interacting with one another. Based on the structure of ubiquitinated PCNA, it is proposed that when pol δ stalls at a DNA lesion, the ubiquitination of PCNA facilitates the recruitment of pol η to the back side of PCNA [126]. The catalytic core of pol η then displaces pol δ since it is connected to the C-terminus of pol η by a long, flexible linker. A recent structural model derived from low-resolution single-particle electron microscopy suggests that pol η can associate with the front face of the PCNA in the editing mode [142]. Additional structures of eukaryotic multi-protein complexes with DNA, PCNA and TLS pols are needed to fully understand how multiple TLS pols are coordinated.

#### 2.2.5. Additional Structural Motifs for Stabilizing PCNA-Pol Complexes

In eukaryotes, B-family DNA polymerases include pol α, pol δ, pol ε, and pol ζ. The former three polymerases are the major players responsible for the bulk of DNA synthesis, and pol ζ is a major error-prone DNA polymerase. In *S. cerevisiae*, pol δ is a three-subunit complex comprised of the catalytic subunit pol3 and accessory subunits pol31 and pol32. The pol δ holoenzyme is formed via interactions between pol31 and the C-terminal segment of pol3, and between pol32 and pol31 [143]. Subunits pol 31 and pol32 are also components of a four-subunit pol ζ4 (discussed in Section 2.4). In addition to the aforementioned structural motifs (PIPs and RIRs) that are important for protein–protein interactions, two conserved cysteine-rich metal-binding motifs (CysA and CysB) within the C-terminal segment of the catalytic subunits of all four B-family DNA polymerases are important for DNA replication and stabilizing multi-protein complexes in *S. cerevisiae* [144]. The Zn-binding motif, CysA of pol3 (the catalytic subunit of yeast pol δ) plays a critical role in PCNA-pol δ complex formation, whereas [4Fe-4S]-binding motif CysB is imperative for the formation of a highly processive yeast pol δ holoenzyme [144]. Mutation of the conserved cysteine residues in the CysA motif significantly decreases the processivity of yeast pol δ; processive DNA replication can be partially restored by adding wild-type pol δ into the system but cannot be restored by adding a mutant form of pol δ without the PIP motif on pol32 (pol32−ΔPIP). By contrast, fully proficient DNA replication was observed for mutant pol δ with pol32−ΔPIP. These results suggest that PIPs may be more relevant for recruiting pols to replication foci in the nucleus, whereas the conserved cysteine-rich metal-binding motifs are important for the formation and/or stability of the PCNA–pol δ complex in processive DNA replication [144]. This is consistent with the previously proposed two-stage recruitment model for TLS polymerases—first, to increase the local concentration of TLS pol(s) at the replication factories, and second, to load TLS pol(s) to the replication termini [38].

### 2.3. Rev1: A Scaffold Protein

*REV1*, along with *REV3* and *REV7*, is among the first translesion synthesis DNA polymerase genes discovered in yeast mutagenesis experiments [4]. Rev1 is the most intriguing Y-family polymerase because of its deoxycytidyl transferase activity [7,88] and its protein template-directed nucleotide incorporation [145]. Yeast genetic studies led to the suggestion that Rev1 has a “second function” separate from its catalytic activity [89]. Subsequent biochemical and cellular studies augmented this proposal by demonstrating that human and mouse Rev1 physically interacts with pol η, pol ι, pol κ, and Rev7 (an accessory subunit of pol ζ) [90,91,92,93], and that the catalytic-null mutant of Rev1 does not affect the levels of mutagenesis induced by DNA-damaging agents [146,147].

#### 2.3.1. Interactions between Rev1 and Other Pols

**Figure 4:**
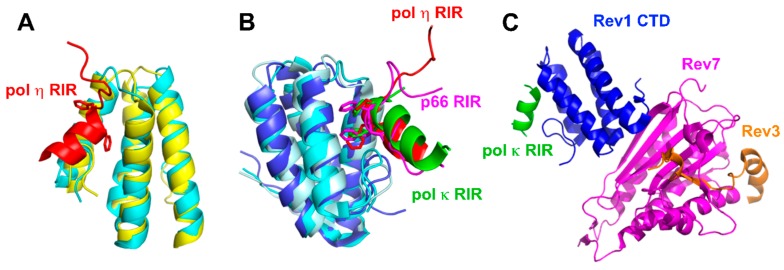
Interactions between Rev1 CTD and the RIR peptides or the interacting fragments of pol ζ. (**A**) Superimposed structures of free human Rev1 CTD (yellow, PDB: 2LSY) and human pol η RIR-bound Rev1 CTD (cyan, PDB: 2LSK). The pol η RIR is in red with the side chains of the conserved phenylalanine residues shown in stick. (**B**) Superimposed structural complexes of mouse Rev1 CTD (blue; PDB:2LSJ) with the pol κ RIR peptide (green), human Rev1 CTD (cyan; PDB:2LSK) with the pol η RIR peptide (red) and human Rev1 CTD (pale cyan; PDB:2N1G) with the p66 (a subunit of pol ζ4) RIR peptide (magenta). Three RIR peptides interact with the same region of Rev1 CTD. The side chains of conserved phenylalanines are shown in stick. (**C**) Mouse Rev1 CTD in complex with Rev7, a fragment of Rev3 and the pol κ RIR peptide (PDB: 4FJO).

The interactions of Rev1 with its protein partners are critically dependent on its C-terminal domain (CTD) [90,91,92,147]. Rev1-interacting proteins contain RIRs that are centered around conserved phenylalanine residues (FF). These interacting proteins include B-family pol δ [110,148,149] and pol ζ [93,150]; Y-family pol ι, pol κ, and pol η [90,91,92,94,119]; base excision repair protein XRCC1 [151]; and yeast Rad5 (a multi-functional protein involved in template switching) [152]. Recent NMR and X-ray crystallographic data have provided a structural basis of the interactions between Rev1 and its partners. According to the solution NMR structures of the mouse Rev1 CTD–pol κ RIR peptide complex and the human Rev1 CTD–pol η RIR peptide complex [153,154], the overall core helix-bundle structure of the RIR-bound human Rev1 CTD is similar to that of the free Rev1 CTD (Figure 4A). Rev1 CTD folds into a four-helix bundle (α1—α4), mediated by a network of interacting residues from individual helices. A majority of these residues are conserved from yeast to human, which contribute to the stability of the CTD of Rev1 across species [154]. Six residues at the N-terminus of α1 helix fold into a structurally defined β-hairpin, and together with the shallow hydrophobic surface between α1 and α2, create a deep hydrophobic cavity for high-affinity binding with RIR peptides [153,154]. The disordered RIR peptides of pol η and κ arrange into a three-turn α-helix upon binding with Rev1 CTD. Two phenylalanine residues of the RIR peptides (of pol η, pol κ, and p66) interact with the hydrophobic cavity of Rev1 CTD (Figure 4B). These two conserved phenylalanine residues are essential for the formation of the protein complex as evidenced by mutational studies in yeast two-hybrid assays [94,153].

#### 2.3.2. Interactions between Rev1 and Pol ζ

Pol ζ is considered as an “extender polymerase” in the generally accepted two-step bypass mechanism in mammals [39,155]. In the first step, an “inserter” polymerase (e.g., pol η, pol ι or pol κ) incorporates a nucleotide opposite the lesion, and in the second step an “extender” polymerase (e.g., pol ζ) extends beyond the base pair that involved the lesion before a replicative polymerase takes over the DNA synthesis. It is well documented that the Rev7 subunit of human pol ζ interacts with Rev1 [93], and that the interaction is functionally important for translesion synthesis across a (6–4) thymine-thymine photoproduct [156]. Since the discovery of a four-subunit complex of pol ζ4 (Rev3-Rev7-p50-p66; p50 and p66 are also subunits of human pol δ) [110,148,149], an additional RIR has been mapped on the p66 subunit of pol ζ, which could also facilitate the formation of Rev1-pol ζ complex [150]. Together, interactions of Rev1 with both Rev7 and p66 potentially contribute to the recruitment of pol ζ via Rev1 and the functional linkage between pol ζ and Rev1.

#### 2.3.3. Coordination of Multiple Binding Partners by Rev1

Recent X-ray crystallographic data have illuminated the molecular mechanisms of the interactions of Rev1 with a number of proteins. Wojtaszek et al. reported a crystal structure of mouse Rev1 CTD in complex with Rev7, an interacting fragment of Rev3 and the pol κ RIR peptide (Figure 4C) [157]. Shortly after, Xie et al. reported the structure of a similar protein complex from humans [158]. In addition, Kikuchi et al. solved the crystal structure of a ternary complex containing the C-terminal domain of human Rev1 CTD, Rev7, and a Rev3 fragment [159]. Collectively, these studies have demonstrated that mammalian Rev1 CTD uses different binding regions to interact with Y-family pols and the Rev7 subunit of pol ζ. As noted earlier, RIRs of pol η and pol κ target the same binding region of Rev1 CTD (Figure 4B), which involves the N-terminal β-hairpin, α1 and α2 helices, and α1-α2 loop [153,154]. On the other hand, Rev7 interacts with a distinct and non-overlapping region of CTD diagonal to the binding site of other Y-family pols (Figure 4C) [157,158], presumably to minimize the chance of steric clash between an “inserter” polymerase and pol ζ at the “insertion” step during Rev1/polζ-dependent TLS [150]. Incidentally, the recently mapped RIR on the p66 subunit of pol ζ interacts with the same site on Rev1 CTD as RIRs of pol η and pol κ do (Figure 4B). Although the dissociation constants (*K*_d_) of Rev1 with RIR peptides vary slightly based on the different techniques used (summarized in Table 2) [94,150,151], RIRs of pol κ and p66 bind to the Rev1 CTD approximately an order of magnitude stronger relative to RIRs of pol ι and pol η. The high affinity between p66 RIR and Rev1 CTD may be a contributing factor to the “inserter” to “extender” polymerase switching in a two-step Rev1/Polζ-dependent TLS [150]. In summary, this body of work has provided structural mechanisms for the interactions between Rev1 and other TLS pols, and such information is important for designing inhibitors to disrupt these interactions [43].

#### 2.3.4. PCNA Tool Belts and Rev1 Bridges

Based on the ways in which TLS polymerases interact with one another and with PCNA, it seems likely that multiple TLS polymerases and PCNA can form higher ordered complexes with different molecular architectures. For example, multiple TLS polymerases can directly interact with PCNA without directly interacting with one another, and form a PCNA tool belt. By contrast, Rev1 can serve as a bridging molecule to link PCNA (via BRCT and/or PAD domains) and another TLS polymerase (via CTD) without PCNA and this other TLS polymerase directly interacting. Such an arrangement is called a Rev1 bridge. Recently, single-molecule studies using yeast PCNA, pol η, and Rev1 have shown that both PCNA tool belts and Rev1 bridges form in approximately equal proportions [160]. Surprisingly, it was observed that these higher ordered complexes were dynamic, meaning that PCNA tool belts can switch to Rev1 bridges and vice versa without dissociation. The dynamic nature of these complexes likely permits rapid sampling of multiple TLS polymerases to find the one that is most appropriate for bypassing a given DNA lesion [160].

#### 2.3.5. Physiological Functions of Rev1-Mediated Protein Interactions

The functional importance of Rev1-mediated protein–protein interactions appears to be polymerase- and lesion-specific. In the case of pol η-mediated CPD bypass, the formation of pol η foci is dependent on the interactions between PCNA and pol η (via PIPs and UBZ of pol η) [125,131], but not on the interactions between Rev1 and pol η (via RIRs) [161]. In keeping with these data, complementation with a variant form of pol η with a F to A mutation in the RIRs resulted in a similar extent of suppression of UV-induced mutagenesis in XP-V fibroblasts relative to cells complemented with wild-type pol η [162]. On the other hand, transient expression of wild-type pol κ in pol κ-knockout mouse embryonic fibroblast cells restored the resistance to BPDE, whereas complementation with pol κ bearing substitutions of phenylalanine residues in RIR fails to correct BPDE-sensitivity [94]. Together, Rev1 plays an important role in interacting with multiple TLS pols, but the biological significance of these interactions remains to be firmly established.

### 2.4. Subunits Sharing between Pol δ and Pol ζ

#### 2.4.1. The Subunit Organization of Pol ζ

In 2012, two groups discovered that yeast pol31 and pol32 proteins (previously recognized subunits of pol δ) together with the Rev3-Rev7 complex of pol ζ form a four-subunit pol ζ4 [110,149]. Similarly, p50 (POLD2) and p66 (POLD3) (human counterparts of yeast pol31 and pol32, respectively) are also components of pol ζ4 in humans [148]. Pol ζ4 has a higher catalytic activity than the minimally functional Rev3-Rev7 complex [110,149,163], and its activity is further enhanced in the presence of PCNA [110]. The pol ζ4 complex is organized via interactions between Rev3 and Rev7, Rev3 and pol31, pol31 and pol32, and pol32 and Rev7 [110,148,149,163]. In addition, pol32 is known to interact with PCNA, which is important for processive DNA replication by pol δ [164]. Analogous to the interaction between pol31 and the C-terminal segment of pol3, CysB of Rev3 (one of the two conserved cysteine-rich metal binding motifs) is essential for Rev3-pol31 interactions [110,148]. A structural model of yeast pol ζ4 based on electron microscopy reconstruction has been reported [165]. In this model, pol ζ4 adopts an elongated bilobal architecture, whereby Rev3 occupies a large lobe of the electron microscopy density map, and accessory subunits (Rev7, pol31, and pol32) locate in a small lobe connected to Rev3 via a longer amino acid linker.

#### 2.4.2. Switching between Pol δ and Pol ζ

Baranovskiy et al. proposed that the subunits sharing between pol δ and pol ζ may be a mechanism to facilitate polymerase switching [148]. Specifically, when pol δ stalls at a DNA lesion, p125 (the catalytic subunit of pol δ) dissociates from p50-p66 for pol ζ to gain access to the replication fork. A caveat is that this proposal does not explain how pol ζ may operate on the leading strand (replicated by pol ε) [148]. Although this proposal remains to be explicitly tested, it provides a basis for further hypothesis generation and testing. Two possible pathways have been postulated for this polymerase switching model [166]. First, the p50-p66 complex remains attached to PCNA to interact with Rev3-Rev7 for pol ζ4 to gain access to the fork. Subunits p125 and p12 (an accessory subunit of pol δ) can be degraded by proteolysis [167]. Second, p50-p66 dissociates from the fork together with p125, and a pre-assembled pol ζ4 complex is recruited for translesion synthesis. The latter pathway is augmented by the observation that p50-p66 complex binds to Rev3 fairly strongly, which withstands stringent washing with 1.0 M NaCl solution [163]. Interestingly, a recent proteomic analysis discovered significant changes of the levels of multiple components of pol δ when comparing wild-type cells to POLD3-deficient mouse cells, and that the levels of pol ζ constituents remain unchanged [168], which implies that p50-p66 may be preferentially associated with pol δ under normal conditions without DNA damage. The concentrations of p50 and p66 at the fork and their preferential association with pol δ or pol ζ under different cellular conditions remain to be determined. Remarkably, Stepchenkova et al. observed that a defect in the catalytic subunit of pol δ that affects the [4Fe-4S] cluster binding leads to suppressed UV-induced mutagenesis and enhanced pol ζ-dependent spontaneous mutagenesis in a yeast strain. On the basis of this finding, the authors proposed that the conserved [4Fe-4S] cluster in pol3 and Rev3 plays a role in pol δ-pol ζ switching [169]. It is imperative to decipher the functional importance of Fe-S clusters in various aspects of DNA metabolism, including polymerase switching, and this question is being actively pursued in the field.

### 2.5. Proteasomal Degradation of DNA Polymerases

#### 2.5.1. Regulation of the Steady-State Levels of TLS Pols

The error-prone nature of TLS polymerases means their access to the replication fork must be carefully regulated. Controlling the steady-state levels of DNA polymerases is a simple way to restrict enzymatic activities of low fidelity DNA polymerases. In *E. coli*, TLS pols are regulated via the global SOS response [42,170,171]. The levels of *E. coli* pol II, pol IV and pol V increase dramatically following LexA inactivation, which contributes to the polymerase switching ([42] and references therein). On the contrary, eukaryotes do not seem to use the overall expression level of TLS pols to respond to genotoxic stress [27], likely due to a larger number of TLS pols in eukaryotes compared to *E. coli*. Nonetheless, the steady-state levels of TLS pols are under strict regulation throughout the cell cycle in eukaryotes. In *S. cerevisiae*, the steady-state levels of both pol η and Rev1 peak at G2/M phase relative to G1 phase and early S phase, whereby a 3-fold increase is observed for pol η and a 50-fold increase is observed for Rev1 [172,173,174,175]. In *Schizosaccharomyces pombe*, Rev1 exists at the highest level in G1 phase and is down-regulated at the entry of S phase of the cell cycle [176]. The exact reason of such regulations remains unknown.

Contradictory results exist regarding whether the overexpression of TLS pols is associated with increased mutagenicity. While King et al. showed no mutagenic effects upon overproducing pol η in diploid XP-V fibroblasts [177], other studies using yeast and mammalian systems demonstrated that overproduction and deletion of *RAD30*/*POLH* result in mutator phenotypes. For instance, overexpression of *POLH* in a multicopy episomal vector has been shown to be toxic to human cells [178]. Abnormal up-regulation of human pol η through IRF1 transactivation leads to an elevated mutation frequency and carcinogenesis in human cells upon exposure to the alkylating agent *N*-methyl-*N′*-nitro-*N*-nitrosoguanidine [179]. When *RAD30* gene is compromised [10,15,180] or overexpressed [181,182] in *S. cerevisiae*, replication infidelity and genomic instability are observed. Similarly, overexpression of Rev1 confers sensitivity to cisplatin in fission yeast [176]. In addition, TLS pols are over-expressed in a number of cancers, which is considered to be a contributing factor to mutagenicity and resistance to chemotherapies [25,43].

#### 2.5.2. Proteasomal Degradation of TLS Pols

TLS regulation can be achieved in part by proteasomal degradation orchestrated by posttranslational modifications. Posttranslational modifications with ubiquitin or ubiquitin-like modifiers play a critical role in the regulation of normal DNA replication and DNA damage tolerance pathways [183,184]. The attachment of ubiquitin to substrates is achieved via an enzymatic cascade by first attaching ubiquitin to an E1 ubiquitin-activating enzyme, then by transfer of ubiquitin to an E2 ubiquitin-conjugating enzyme, and by finally binding of E2 and substrate together with an E3 ubiquitin ligase, which completes the ubiquitin transfer from the E2 enzyme to the substrate [183]. There have been several reports of different E3 ligases being involved in the ubiquitination of pol η, which include Pirh2 (RING-H2 type E3 ligase) [185,186], mdm2 (murine double minute) [187], TRIP (human TNF receptor associated factor (TRAF)-interacting protein) in humans [188], and NOPO (homolog of human TRIP) in *Drosophila* [188]. For example, Pirh2 physically interacts with and monoubiquitinates human pol η and is involved in the 20S proteasomal degradation of pol η [185,186]. Mdm2 physically interacts with pol η in vivo and in vitro and facilitates pol η degradation via ubiquitin-dependent proteolysis [187]. On the other hand, TRIP and NOPO E3 ligases promote the ubiquitination of pol η, and enhance the localization of pol η in replication foci [188]. Apparently, unlike *E. coli*, eukaryotes prefer to regulate the local concentrations of pols at the fork by modulating the interactions of TLS pols with multiple binding partners. It should be kept in mind that the proteosomal degradation of TLS pols does not necessarily indicate their activities at the replication factories, and whether the degradation targets the soluble pool or chromatin-bound TLS pols remains to be elucidated. Nonetheless, a decrease in the concentration of a given TLS pol is likely to limit its access to the replication fork or to facilitate its removal after TLS.

#### 2.5.3. Protein Degradation Creates Binding Sites for TLS Pols

CRL4^Cdt2^ (*C*ullin 4-*R*ING *L*igase (CRL4)-Ddb1-Cdt2) is an E3 ubiquitin ligase that targets PCNA binding partners for proteasomal degradation and is known as a master regulator for genomic stability [189]. CRL4^Cdt2^ mediates the degradation of replication licensing factor Cdt1, which prevents DNA re-replication and genome instability [189]. In addition, CRL4^Cdt2^ facilitates the rapid degradation of Cdt1 after DNA damage [190,191]. In *Caenorhabditis elegans*, CRL4^Cdt2^ participates in the degradation of pol η [192]; however, whether CRL4^Cdt2^ is involved in the degradation of pol η in humans is yet to be tested. A number of CRL4^Cdt2^ substrates including Cdt1 contain specialized PIP modules (PIP degrons), which are important for protein degradation [189,193,194]. Compared to a canonical PIP sequence, a PIP degron contains both a TD motif and a basic amino acid four residues downstream ([Q/N]xxφTD[F/Y][F/Y]xxx[R/K]); the conserved TD motif confers stronger PCNA binding relative to canonical PIPs [193,194]. The conserved threonine residue within the Cdt1 PIP degron is important for interfering with pol η foci formation after UV damage [195]. Importantly, CRL4^Cdt2^-mediated proteolysis facilitates pol η and pol κ focus formation after UV-induced DNA damage [195]. Thus, it is proposed that CRL4^Cdt2^-mediated Cdt1 degradation unmasks the site on PCNA for the binding of TLS pols [195], although the molecular basis of this model remains to be established.

#### 2.5.4. Proteasomal Degradation of Pol δ

Protein degradation is an important means to regulate multi-subunit replicative DNA polymerase δ, which potentially contributes to the displacement of pol δ at a stalled fork. Human pol δ is a four subunit complex (p125-p50-p66-p12, herein after referred to as pol δ4) [196]. Collective studies by Lee and colleagues have shown that the p12 subunit of pol δ holoenzyme is subject to rapid proteolysis in human cells triggered by DNA damage or replication stress [167]. The loss of p12 leads to the formation of a trimeric form of pol δ3 (p125-p50-p66), which has impaired catalytic activities relative to pol δ4 [167,196]. Detailed kinetic characterizations revealed that such a compromise in catalytic activity is mainly attributed to a decreased burst rate (a function of the rates of phosphodiester bond formation and conformational change) and a greater proofreading activity of pol δ3 [197]. As a result, pol δ3 has an increased tendency to stall at DNA lesions, which may facilitate the exchange of TLS pols [198]. Interestingly, subsequent studies indicate that pol δ3 also functions during unperturbed DNA replication [199,200], and the level of p12 subunit remains at a baseline level during unperturbed growth in unsynchronized cells [201]. As the authors pointed out, these studies measure the nuclear pool of p12 and pol δ3, and do not provide direct information on the assembly of pol δ at the replication fork [199]. Therefore, future studies are needed to fully understand the biological functions of pol δ3 and pol δ4, as well as the partition between the two. A recent study by Hedglin et al. demonstrates that human pol δ4 maintains a loose association with PCNA when replicating DNA, and that pol δ4 holoenzyme is relatively unstable and rapidly dissociates upon stalling [202]. These authors suggest that on a lagging strand it may not be necessary for polymerases to engage in active polymerase switching in humans [128]. It is likely that p12 maintains a dynamic equilibrium between association and dissociation during lagging strand DNA synthesis, especially considering that pol δ has to continually replace pol α at primed sites [203,204].

## 3. Concluding Remarks

In summary, the understanding of the selection and switching of DNA polymerases has substantially advanced over the past decade. Nonetheless, questions remain regarding the molecular mechanisms of these processes. First, structures of multi-protein complexes with one or more specialized DNA polymerases, DNA, PCNA and a replicative DNA polymerase need to be solved. Such structures will be useful to further understand the coordination of multiple factors at the fork. Although protein complexes are often recalcitrant for crystallization, recent advances in cryo-electron microscopy holds promise for solving the problem. Second, the dynamics of multi-protein assembly remain poorly understood. Single molecule techniques together with rapid kinetics can potentially tackle this problem. Third, novel approaches are needed to systematically understand the coordination of multiple components during the selection and switching of DNA polymerases. Modern omics-based approaches in combination with bioinformatics may offer new solutions to this challenging task.

## Figures and Tables

**Table 1:** Apparent dissociation constants (*K*_d_) of DNA polymerase holoenzymes or PIP peptides with PCNA. Conserved amino acid residues relative to a consensus sequence are in bold. Italic cysteines indicate that these amino acid residues were included in addition to the native RIR peptide to facilitate the measurement.

DNA Polymerase	Sequence	*K*_d_ (μM)
pol δ PIP	_451_GKANR**Q**VS**I**TG**FF**QRK	16 ^1^
pol δ holoenzyme		<0.010 ^2^
pol η PIP2	*C*_694_KRPRPEGMQT**L**ES**FF**KPLTH	0.40 ^3^
pol η holoenzyme		0.12 ^4^
pol κ PIP + PLTH	*C*_856_IKPNNPKHT**L**DI**FF**K*PLTH*	4.9 ^3^
pol ι PIP	*C*_419_AKKGL**I**DY**Y**LMPSLST	0.39 ^3^

^1^ Measured by isothermal titration calorimetry [112]; ^2^ Estimated using a binding assay containing forked DNA-PCNA complex as substrate and pol δ as ligand. Values in ^2^ and ^4^ are from ref. [121]; ^3^ Obtained from surface plasmon resonance (SPR) assays [114].

**Table 2:** Apparent dissociation constants (*K*_d_) of human p66 (a subunit of pol δ), pol η, pol κ, and pol ι RIR peptides with human Rev1. Conserved phenylalanine residues are in bold.

DNA Polymerase	Sequence	*K*_d_ (μM) SPR	Fluorescence ^3^
p66	_231_KGNMMSN**FF**GKAAMNK	2.3 ^1^	
pol η	_524_QSTGTEP**FF**KQKSLLL	13 ^2^	4.4
pol κ	_560_EMSHKKS**FF**DKKRSER	7.6 ^2^	
pol κ	_560_EMSHKKS**FF**DKKRSER	1.7 ^1^	0.28
pol ι PIP	_539_ASRGVLS**FF**SKKQMQD	69 ^2^	5.5

^1,2^ Values are from references [94,150], respectively, and are obtained with surface plasma resonance (SPR) assays; ^3^ Calculated from fluorescence titration assays [151].
